# Experimental Infection and In-Contact Transmission of H9N2 Avian Influenza Virus in Crows

**DOI:** 10.3390/pathogens11030304

**Published:** 2022-02-28

**Authors:** Asha Kumari Verma, Manoj Kumar, Harshad V. Murugkar, Shanmugasundaram Nagarajan, Chakradhar Tosh, Pushpendra Namdeo, Rupal Singh, Suman Mishra, Subbiah Kombiah, Senthilkumar Dhanapal, Vijendra Pal Singh

**Affiliations:** ICAR-National Institute of High Security Animal Diseases, OIE Reference Laboratory for Avian Influenza, Anand Nagar, Bhopal 462022, Madhya Pradesh, India; ashav223@gmail.com (A.K.V.); harshadmurugkar@gmail.com (H.V.M.); nagavetbio@gmail.com (S.N.); chakradhar.tosh@gmail.com (C.T.); pushpendranamdeo30@gmail.com (P.N.); rupalsingh86@gmail.com (R.S.); sumanrewa121@gmail.com (S.M.); kombiahmaruthu@gmail.com (S.K.); senvetpath@gmail.com (S.D.); vijendra61@gmail.com (V.P.S.)

**Keywords:** avian influenza, H9N2 virus, crows, infection, in-contact transmission, zoonoses

## Abstract

This study aimed to investigate the potential of H9N2 avian influenza virus to cause disease and intra-species transmission in house crows (*Corvus splendens*). A group of six crows were intranasally inoculated with 10^6.0^ EID_50_ of H9N2 virus (A/chicken/India/07OR17/2021), and 24 h post-inoculation six naïve crows were co-housed with infected crows. Crows were observed for 14 days for any overt signs of illness. Oropharyngeal and cloacal swabs were collected up to 14 days to assess virus excretion. No apparent clinical signs were observed in either infected or in-contact crows. Virus excretion was observed only in infected birds up to 9 days post-infection (dpi) through both oropharyngeal and cloacal routes. All six infected crows seroconverted to H9N2 virus at 14 dpi, whereas all in-contact crows remained negative to H9N2 virus antibodies. No virus could be isolated from tissues viz., lung, liver, kidney, pancreas, small intestine and large intestine. Although crows became infected with the H9N2 virus, transmission of the virus was inefficient to the in-contact group. However, virus excretion through oral and cloacal swabs from infected crows suggests a potential threat for inter-species transmission, including humans. Crows, being a common synanthrope species, might have some role in influenza virus transmission to poultry and humans, which needs to be explored further.

## 1. Introduction

Influenza A viruses belong to the *Orthomyxoviridae* family and are characterized based on the combinations of their surface glycoproteins, haemagglutinin (HA) and neuraminidase (NA). So far, 16 HA and 9 NA genetically distinct subtypes have been identified in birds, while H17N10 and H18N11 subtypes have been reported only in bats [1,2]. Low pathogenic avian influenza (LPAI) H9N2 virus is of a particular public health concern due to its widespread circulation in domestic poultry throughout Eurasia and Africa [3,4,5]. Since the first report of the H9N2 influenza virus in turkeys in Wisconsin in 1966 [6], it has evolved as one of dominant influenza virus subtypes circulating in poultry and wild birds. Prior or concurrent infection with H9N2 viruses masks the clinical disease, contributing to the spread of highly pathogenic avian influenza (HPAI) viruses [5,7].

Genetic analysis of H9N2 avian influenza viruses has shown that they are associated with some zoonotic spillover episodes leading to emergence of extensive reassortment viruses with other co-circulating subtypes of avian influenza viruses, including HPAI H5N1 and H7N3 viruses [8,9]. Furthermore, some LPAIV H9N2 isolates have shown affinities for the SA-2, 6 receptors, which has been linked to the Q226L amino acid mutation in the HA gene, causing mammalian adaptation [10,11]. These mutations have permitted the H9N2 virus to cross the intra-species barrier to cause sporadic human infections [12]. Many factors influence the spread of these novel reassortant viruses, but the efficiency of transmission within and across susceptible species is crucial.

Wild migratory birds are known to harbor all possible subtypes of avian influenza viruses [13], contributing to their geographical dissemination and maintenance [14,15]. Due to the low possibility of contact, direct transmission between wild migratory birds and human populations is uncommon; however, some synanthropic birds (such as house sparrows and crows) might play some role in influenza virus transmission.

Earlier studies have also described the infectivity and transmissibility of various LPAI and HPAI viruses in different poultry and other wild bird species [16,17,18]; however, marked differences in infectivity and transmissibility have been observed between different strains within subtypes [19], and therefore data from one subtype cannot be extrapolated to other subtypes or strains.

Crows (*Corvus splendens*) are a common synanthropic species that share habitats with wildlife, poultry, and humans, and may serve as bridge hosts facilitating spillover from aquatic bird hosts to poultry, as well as humans. HPAI H5 avian influenza virus infection causes and exhibits clinical signs related to the affection of the nervous system, such as wing paralysis, torticollis, etc., and mortality in crows [20,21,22]. While there are no reports of natural outbreak, seropositivity for H9N2 virus has been reported in crows [23,24], suggesting milder infection.

To date, limited information is available on the infection and transmission of the H9N2 virus in crows. Herein, we examined the infection and in-contact transmission of the H9N2 virus in crows to explore the role of crows as carriers or reservoirs of H9N2 infection to poultry and humans. 

## 2. Result

### 2.1. Clinical Signs

There were no apparent clinical signs observed in either the infected or the in-contact groups of crows. Feed intake in infected crows reduced at 2 days post inoculation (dpi), and resumed back to normal on 4 dpi. The cloacal temperature remained within a normal range of 40.5–41 °C in infected as well in in-contact crows, up to 14 dpi. All four crows in the control group remained healthy throughout the experiment.

### 2.2. Virus Shedding 

Analysis of oropharyngeal and cloacal swabs, taken from 1–14 dpi from each bird, revealed that infected birds were positive for the virus, whereas no virus was detected from in-contact birds. Viral isolation and viral quantification from swabs and tissues are summarized in Table 1 and Table 2. Viral isolation was observed only after two serial passages in ECEs, except for one oral swab. The virus was isolated from both oropharyngeal and cloacal swabs through 2 to 7 dpi, but could no longer be isolated on subsequent days; however, viral RNA could be detected up to 9 dpi by qRT-PCR. Only a small proportion of virus was isolated from the qRT-PCR positive swabs. Viral shedding was inconsistent in the infected group from the 1st to 9th dpi (RNA copies ranged between 4.1 × 10^4^–7.7 × 10^8^/mL); however, maximum shedding was observed from 2 dpi to 4 dpi. The highest viral RNA copies were detected on 3 dpi in both oropharyngeal and cloacal swabs (7.7 × 10^8^ and 1.0 × 10^8^ RNA copies/mL, respectively). Low viral RNA copies were detected in tissues of infected crows euthanized at 14 dpi (Table 3). The virus was not detected in any of the swabs or tissues collected from in-contact birds.

### 2.3. Serology

The presence of antibodies against H9N2 avian influenza virus was observed at the 7th and 14th dpi. At the 7th dpi, two out of six infected crows were positive for haemagglutination inhibition (HI) antibodies, whereas at 14 dpi, all six infected crows seroconverted to H9N2 infection (Table 4). All in-contact and control group birds remained negative to H9N2 virus antibodies.

### 2.4. Sequence Analysis of HA Gene 

The HA gene of the A/chicken/India/07OR17/2021 (H9N2) virus was sequenced, and the sequence was deposited in GENBANK (Accession number OM510338). The HA gene showed the highest genetic similarity (92.0% homology) to the H9N2 virus (A/chicken/India/3/2003(H9N2) of G1-like sublineage from India, isolated during 2003. Sequence analysis revealed an insertion of alanine aminoacid at position 12 with a human receptor (α 2,6 sialic acid) specificity motif (^235^LIG^237^) and the absence of multiple basic amino acids at the HA cleavage site (^336^KSSR*GLF^342^). 

## 3. Discussion

Low pathogenic H9N2 viruses, unlike HPAI viruses, cause low or even no clinical disease, and are therefore gradually overlooked; however, they have become well established in different species of poultry. As there is no systemic disease, surveillance studies for the prevalence of H9N2 viruses have been completed, with several studies showing prevalence of H9N2 viruses in different poultry and wild birds [5,12]. Indian house crows (*Corvus splendens*) are ubiquitous throughout the country, and wholly dependent on human habitation for their food. They have small distance migration which leads to frequent contact with human habitation where poultry live in close proximity. Apart from this, they can carry part of or/whole carcasses from place to place. They are expected to play a role in the spread of several viruses, such as avian paramyxovirus, avian influenza virus, West Nile disease virus, and prions [25,26].

In our study, we assessed the infection and transmission capacity of the LPAI H9N2 virus from experimentally infected crows to healthy in-contact birds. We observed that the H9N2 virus was not able to cause clinical disease in crows; however, viral shedding through oropharyngeal and cloacal swabs from infected crows was recorded in the present experiment. The shedding was inconsistent and observed from the first to ninth dpi through qRT-PCR. 

All the infected birds seroconverted against H9N2 infection at 14th dpi, suggesting the establishment of infection. The virus was not detected in swabs collected from the in-contact crows, and no seroconversion was observed. Our results concurred with the findings of Iqbal et al. (2013), who also reported non-transmission to in-contact crows and virus excretion in oral and cloacal swabs up to 7 days post-infection in infected crows [27]. However, they did not quantify viral RNA, as they failed to isolate the virus from the buccal and cloacal swabs in the first passage [27]. In the present study, we could isolate the virus in the first passage of the oropharyngeal swab from one crow (Table 1). Genetic analysis of the HA gene of the A/chicken/India/07OR17/2021(H9N2) virus used for this study indicated that the HA gene shares only 89% homology with the Pakistan virus, and an insertion of three nucleic acids ^45^GGC^47^ and one amino acid Alanine at position 12, compared to the Pakistan virus. The correlation between the isolation of the virus in the first passage of oropharyngeal swab from a crow and the genetic differences between the two viruses need further investigation. Many studies reported that sudden mortality in house crows appeared to be an indicator of the circulation of HPAI (H5N1) viruses in domestic poultry [28,29,30]. However, there have been no reports of outbreaks of the H9N2 virus in crows so far. Hence, the role of crows as reservoirs or spillover hosts of avian influenza virus subtypes has not yet been established.

Continuous increase in human cases with H9N2 viruses over the years highlights the need to closely monitor the transmission events occurring at intra and inter species levels. The genetic analysis of recent H9N2 sequences revealed adaptive mutations from avian-like to human-like receptor binding, indicating the capacity to readily infect new hosts without prior adaptation [31,32]. Most of the H9N2 human infections were reported simultaneously with H9N2 influenza virus detection in local poultry, and were mainly due to close contact with poultry [33,34,35]. Seromonitoring of synanthropic species such as crows and sparrows may be useful to understand their role in avian influenza ecology.

Our study indicates that crows can become infected with the H9N2 virus, and oropharyngeal and cloacal shedding suggests a potential threat for inter-species transmission, including humans, which can be further explored. Recent studies have suggested evolutionary changes in the H9N2 virus, making it more adaptable to various hosts; therefore, the data obtained from this study cannot be inferred for other H9N2 viruses. Furthermore, experimental studies generating reassortant avian influenza viruses are warranted to assess the role of peridomestic birds in the transmission of influenza viruses in different poultry species. To conclude, our study adds new insights to rationally assess the risk of transmission of newly evolving H9N2 viruses from infected crows, which have the potential for significant public health implications.

## 4. Material and Methods

### 4.1. Virus

Low pathogenic avian influenza H9N2 virus (A/chicken/India/07OR17/2021), accessed from the repository of ICAR-National Institute of High Security Animal Diseases (NIHSAD), Bhopal, was used in this study.

### 4.2. Experimental Crows

House crows (*Corvus splendens*) were procured from the local market of Bhopal and kept in quarantine for 3 weeks before the start of experiment. Serum, oropharyngeal and cloacal swab samples were collected from all birds. Serum was tested against type A influenza antibodies by enzyme-linked immuno-sorbent assay (ELISA) (IDEXX AI MultiS-Screen Ab Test, Westbrook, ME, USA) and H5 and H9 antibodies by haemagglutination inhibition (HI) using standard methods [36]. Swabs were further processed for virus isolation in 9–11-day-old embryonated chicken eggs (ECE) [36], and viral RNA detection using matrix (M) gene real-time reverse transcription–PCR (qRT-PCR), as described [37], to detect any active influenza virus infection.

### 4.3. Experimental Design

All experiments were performed in the animal containment facility (ABSL3) animal wing of ICAR-NIHSAD, Bhopal. The animal experiments were approved by the Institute Animal Ethics Committee (IAEC) (114/IAEC/NIHSAD/20). The crows were divided into two groups, an infected and an in-contact group containing 6 birds each, with an uninfected group containing four birds serving as control. Each crow in the infected group was intranasally inoculated with 100 µL of 10^6^ EID_50_ virus (A/chicken/India/07OR17/2021) and 24 h post-infection the naïve birds of the in-contact group were housed with them in the same cage. All the crows were monitored daily for clinical signs, such as respiratory distress, ruffled feathers, sneezing, ocular and nasal discharge, wing paralysis, torticollis, and circling movements. Cloacal and oropharyngeal swabs were collected from each crow till the death of the birds or up to the 14th dpi. Blood samples were collected from all infected and in-contact birds on the 7th and 14th dpi. On the 14th dpi, all crows were humanely euthanized and tissues viz. brain, lungs, trachea, spleen, liver, intestine, and kidneys were collected for virus isolation and quantification.

### 4.4. Virus Isolation

Virus isolation from swabs and tissues was performed by inoculation into 9–11-day-old ECE, and confirmation of virus in the allantoic fluids was determined by virus haemagglutination (HA) and haemagglutination inhibition assays using standard procedures [36]. 

### 4.5. HI Test

Blood samples collected before the start of the experiment and on the 7th and 14th dpi were tested for determination of HI antibody titres against H9N2 virus, using the standard HI test [36].

### 4.6. Viral RNA Quantification

Viral RNA was extracted from swabs using Nucleospin RNA extraction kit (Macherey-Nagel, Düren, Germany) and from tissues using TRIzol® Reagent (Ambion, Life Technologies, Austin, TX, USA), respectively. RNA was quantified by using Qubit® Fluorometer using Quant-iT™ RNA Assay Kit (Invitrogen, Waltham, MA, USA) as per the manufacturer’s protocol. In vitro transcription (IVT) synthesis was used to generate standard RNA from a matrix clone obtained from the repository at NIHSAD using mMESSAGE mMACHINE™ T7 Transcription Kit (Invitrogen). Viral RNA copy number was calculated by the formula: (concentration of RNA in grams/μL/ [length of amplicon × 340 × 109]) × 6.022 × 1023 = Number of molecules/μL). Then, qRT-PCR was performed to obtain a standard curve using serial ten-fold dilution of the IVT RNA [37], and viral RNA copy number was calculated from C_t_ value of samples. 

### 4.7. Haemagglutinn Gene Characterisation

Viral RNA was extracted from 140 µL of infected allantoic fluid using the ‘QIAamp Viral RNA mini kit’ (Qiagen, Hilden, Germany) as described by the manufacturer, and the HA gene was amplified, as described in an earlier study [10]. The PCR products were sequenced using a BigDye Terminator v3.1 cycle sequencing kit on an ABI 3130 genetic analyzer (Applied Biosystems, Bedford, MA, USA), following the manufacturer’s guidelines. The sequences were assembled and edited using the Lasergene 99 program package (DNASTAR, Madison, WI, USA).

## Figures and Tables

**Table 1:** Number of HA positive swabs collected over 14 days from infected and in-contact crows.

Group	No. of Birds	Passage Number	Swab Sample	Day Post Infection						
				2	3	4	5	6	7	8 to 14
Infected	6	1st	OR	–	1(5)	–	–	–	–	–
			CL	–	–	–	–	–	–	–
		2nd *	OR	3 (3.9 ± 0.3) **	2 (3.1 ± 0.47) **	3 (3.6 ± 0.3) **	2 (3.5 ± 0.28) **	1 (4) **	1 (4) **	–
			CL	2 (3.9 ± 0.28) **	3 (4.1 ± 0.55) **	2 (4 ± 0.4) **	1 (3) **	2 (3.5 ± 0.28) **	–	–
Contact	6	1st	OR	–	–	–	–	–	–	–
			CL	–	–	–	–	–	–	–
		2nd	OR	–	–	–	–	–	–	–
			CL	–	–	–	–	–	–	–

OR, denotes oropharyngeal swabs. CL, denotes cloacal swabs. * Swabs from crows showed virus presence after two serial passages in eggs. ** Mean log_2_ HA titres.

**Table 2:** Viral RNA quantification (copies/gm sample) of tissue samples obtained from infected crows.

Crow No.	Brain	Trachea	Lung	Heart	Liver	Kidney	Spleen	Pancreas	Small Intestine	Large Intestine
1	–	–	–	–	–	–	–	–	2.3 × 10^5^	–
2	–	–	–	–	–	7.3 × 10^5^	8.7 × 10^4^	1.0 × 10^5^	1.9 × 10^6^	–
3	–	–	–	–	–	–	–	8.1 × 10^4^	1.3 × 10^6^	–
4	–	–	–	–	–	2.6 × 10^5^	1.7 × 10^5^	–	–	–
5	–	–	–	–	–	–	–	–	–	–
6	3.4 × 10^4^	–	8.0 × 10^5^	–	7.3 × 10^5^	–	–	–	–	5.8 × 10^4^

**Table 3:** Viral RNA quantification (copies/mL sample) of oropharyngeal (OR) and cloacal (CL) swabs obtained from infected crows.

Crow No.	Swabs	Day Post Infection								
		1	2	3	4	5	6	7	8	9
1	OR	2.0 × 10^6^	5.8 × 10^6^	7.7 × 10^8^	4.2 × 10^6^	–	2.9 × 10^5^	2.0 × 10^5^	–	–
	CL	1.0 × 10^6^	2.5 × 10^6^	2.3 × 10^7^	9.9 × 10^6^	–	5.9 × 10^6^	8.8 × 10^4^	–	–
2	OR	2.4 × 10^6^	1.8 × 10^6^	4.6 × 10^7^	1.4 × 10^6^	7.0 × 10^7^	1.5 × 10^5^	9.0 × 10^4^	1.1 × 10^6^	–
	CL	2.7 × 10^6^	1.1 × 10^6^	1.0 × 10^8^	1.1 × 10^6^	6.4 × 10^6^	3.5 × 10^6^	–	–	–
3	OR	1.9 × 10^6^	4.1 × 10^4^	1.8 × 10^7^	1.0 × 10^6^	7.8 × 10^6^	2.1 × 10^6^	2.6 × 10^6^	–	–
	CL	1.2 × 10^6^	4.5 × 10^5^	2.1 × 10^7^	5.6 × 10^6^	9.8 × 10^5^	–	–	–	–
4	OR	3.0 × 10^6^	1.8 × 10^6^	–	4.5 × 10^5^	–	–	1.3 × 10^6^	–	6.6 × 10^5^
	CL	7.6 × 10^6^	6.6 × 10^6^	–	1.1 × 10^8^	–	–	1.9 × 10^6^	–	–
5	OR	–	3.2 × 10^6^	1.9 × 10^7^	1.7 × 10^6^	1.1 × 10^6^	–	2.2 × 10^7^	–	–
	CL	–	6.0 × 10^5^	1.5 × 10^7^	5.1 × 10^6^	–	4.7 × 10^6^	–	–	–
6	OR	2.6 × 10^5^	2.2 × 10^5^	1.0 × 10^7^	5.7 × 10^7^	4.5 × 10^5^	4.3 × 10^6^	–	–	–
	CL	1.8 × 10^6^	1.9 × 10^7^	2.8 × 10^7^	2.2 × 10^7^	–	7.5 × 10^5^	–	–	–

**Table 4:** Seropositivity in infected and contact birds.

Group	Pre-Infection HI Titre	Number of Birds Positive for HI/Total Birds	
		7 Dpi	14 Dpi
Infected	<2 **	2/6 (4.47 ± 0.5) *	6/6 (4.9 ± 0.3) *
Contact	<2 **	0/6	0/6

Dpi, days post infection. * Mean log_2_ HI titres. ** HI titres < 2 log_2_ were considered seronegative.

## Data Availability

Data is contained within the article.
